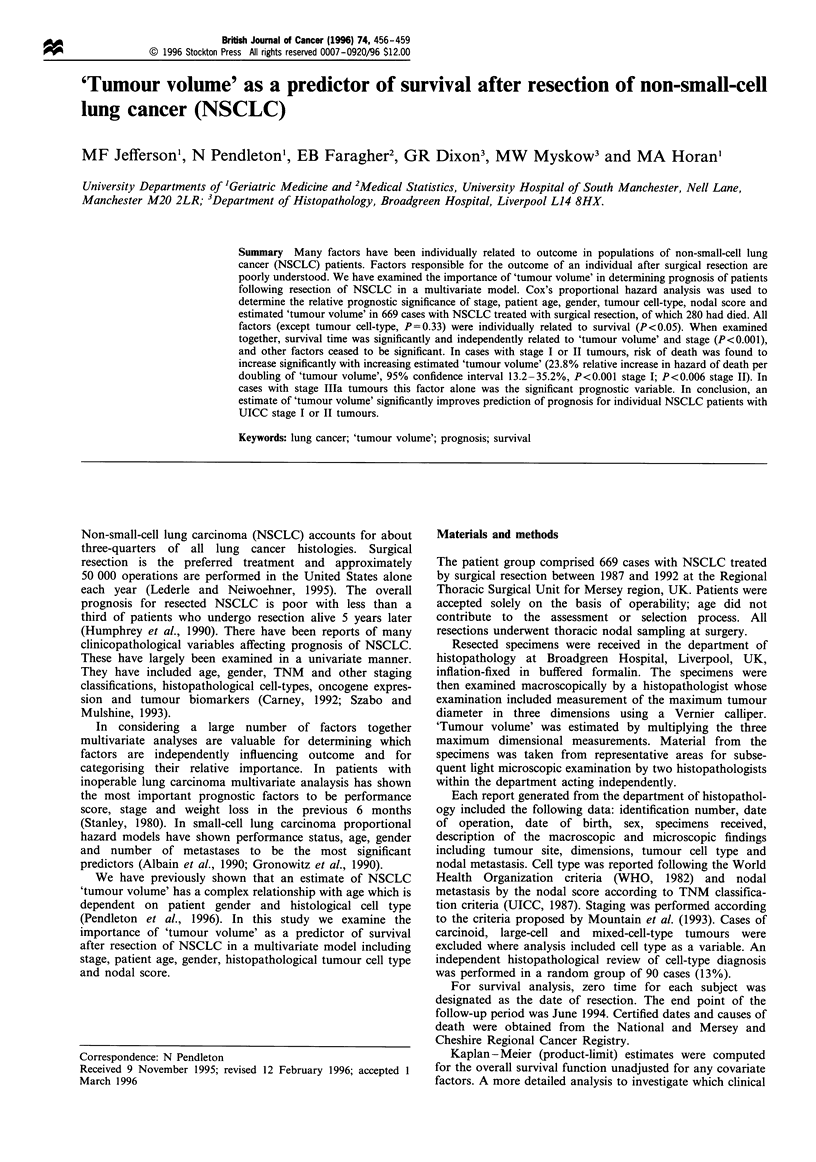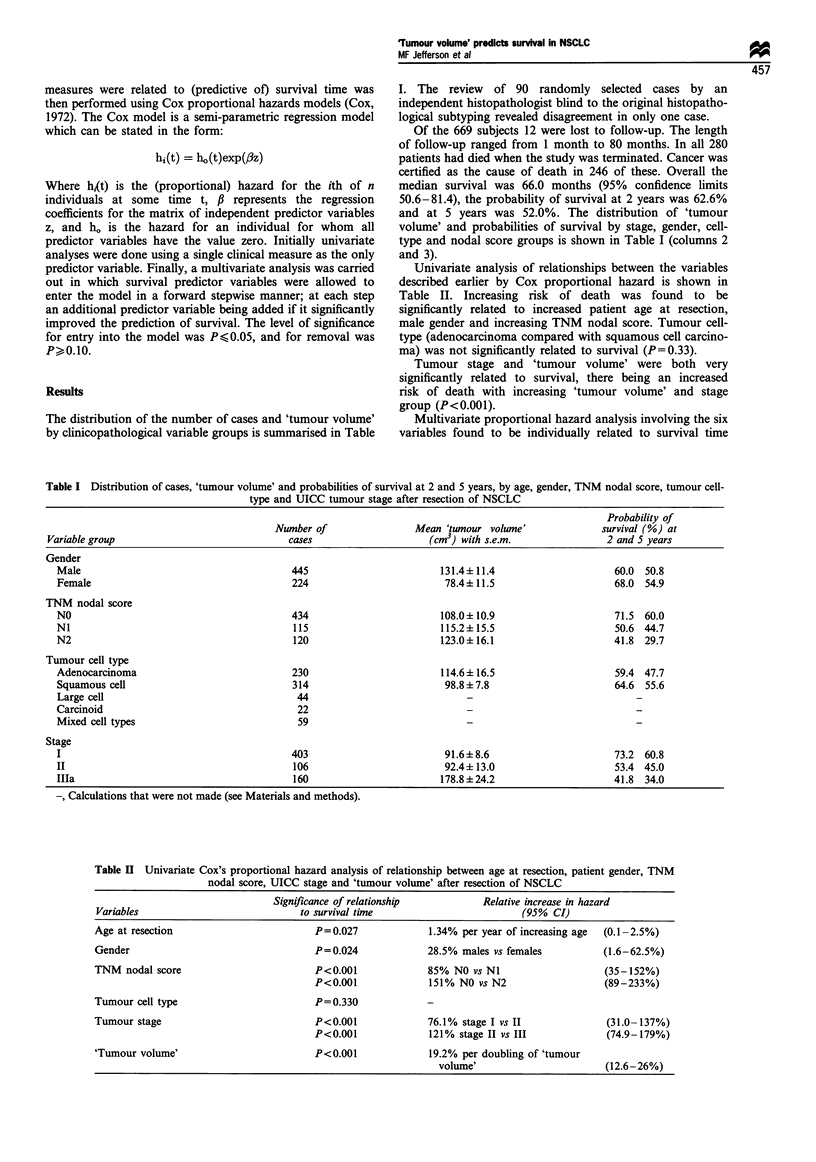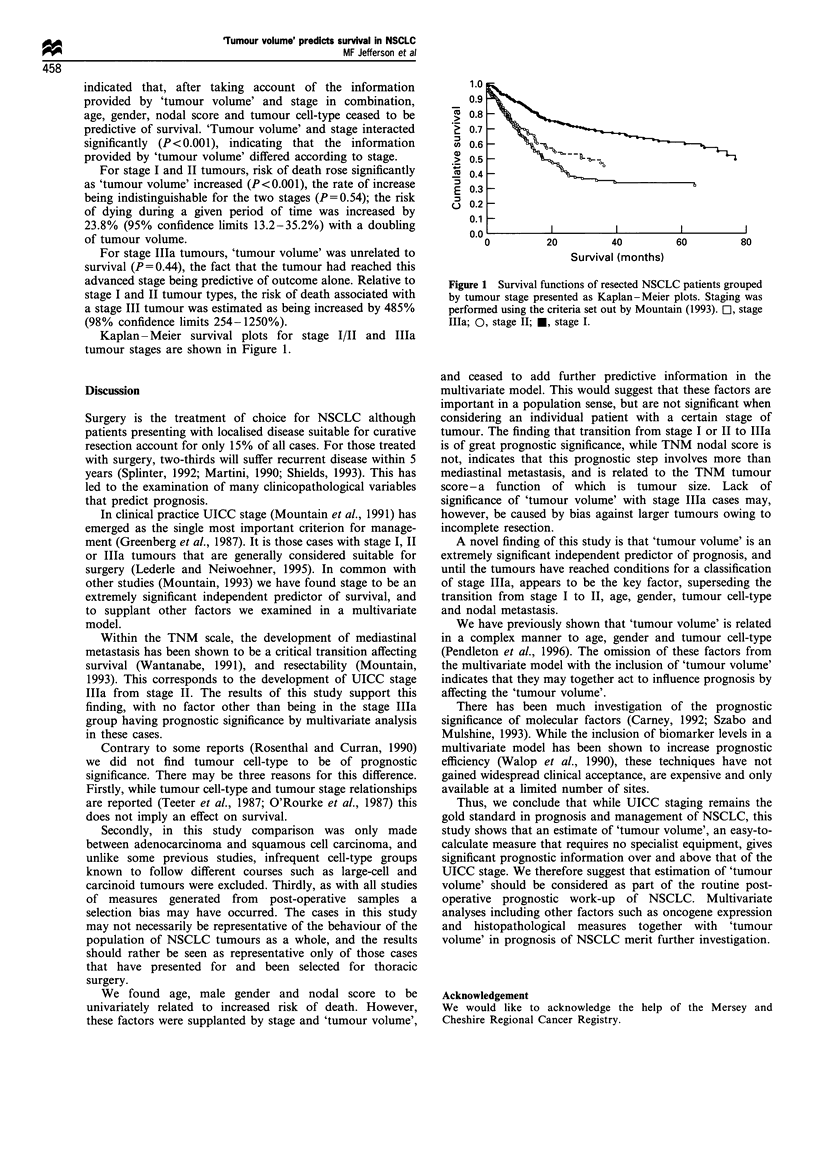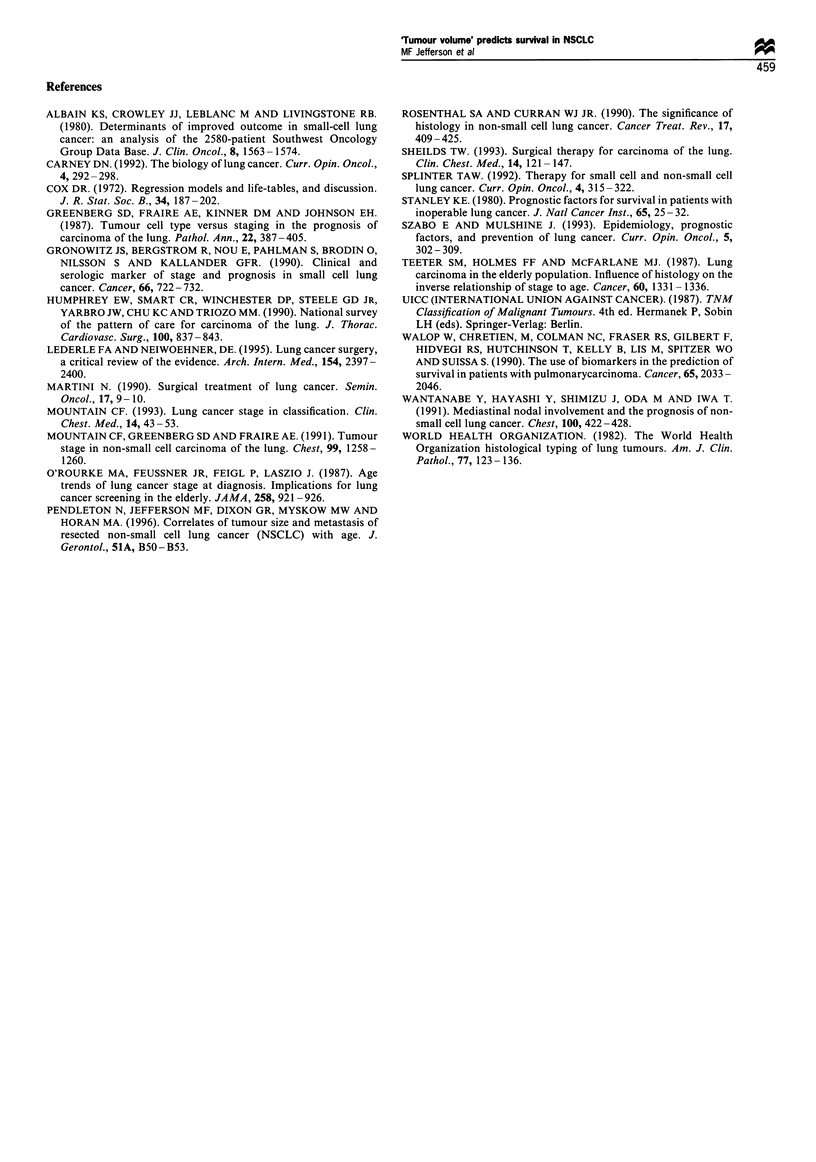# 'Tumour volume' as a predictor of survival after resection of non-small-cell lung cancer (NSCLC)

**DOI:** 10.1038/bjc.1996.381

**Published:** 1996-08

**Authors:** M. F. Jefferson, N. Pendleton, E. B. Faragher, G. R. Dixon, M. W. Myskow, M. A. Horan

**Affiliations:** University Department of Geriatric Medicine, University Hospital of South Manchester.

## Abstract

Many factors have been individually related to outcome in populations of non-small-cell lung cancer (NSCLC) patients. Factors responsible for the outcome of an individual after surgical resection are poorly understood. We have examined the importance of 'tumour volume' in determining prognosis of patients following resection of NSCLC in a multivariate model. Cox's proportional hazard analysis was used to determine the relative prognostic significance of stage, patient age, gender, tumour cell-type, nodal score and estimated 'tumour volume' in 669 cases with NSCLC treated with surgical resection, of which 280 had died. All factors (except tumour cell-type, P = 0.33) were individually related to survival (P < 0.05). When examined together, survival time was significantly and independently related to 'tumour volume' and stage (P < 0.001), and other factors ceased to be significant. In cases with stage I or II tumours, risk of death was found to increase significantly with increasing estimated 'tumour volume' (23.8% relative increase in hazard to death per doubling of 'tumour volume', 95% confidence interval 13.2-35.2%, P < 0.001 stage I; P < 0.006 stage II). In cases with stage IIIa tumours this factor alone was the significant prognostic variable. In conclusion, an estimate of 'tumour volume' significantly improves prediction of prognosis for individual NSCLC patients with UICC stage I or II tumours.


British Journal of Cancer (1996) 74, 456-459
© 1996 Stockton Press All rights reserved 0007-0920/96 $12.00

### 'Tumour volume' as a predictor of survival after resection of non-small-cell lung cancer (NSCLC)

MF Jefferson[1], N Pendleton[1], EB Faragher[2], GR Dixon[3], MW Myskow[3] and MA Horan[1]

*University Departments of [1]Geriatric Medicine and [2]Medical Statistics, University Hospital of South Manchester, Nell Lane, Manchester M20 2LR; [3]Department of Histopathology, Broadgreen Hospital, Liverpool L14 8HX.*

**Summary** Many factors have been individually related to outcome in populations of non-small-cell lung cancer (NSCLC) patients. Factors responsible for the outcome of an individual after surgical resection are poorly understood. We have examined the importance of 'tumour volume' in determining prognosis of patients following resection of NSCLC in a multivariate model. Cox's proportional hazard analysis was used to determine the relative prognostic significance of stage, patient age, gender, tumour cell-type, nodal score and estimated 'tumour volume' in 669 cases with NSCLC treated with surgical resection, of which 280 had died. All factors (except tumour cell-type, $P=0.33$) were individually related to survival ($P<0.05$). When examined together, survival time was significantly and independently related to 'tumour volume' and stage ($P<0.001$), and other factors ceased to be significant. In cases with stage I or II tumours, risk of death was found to increase significantly with increasing estimated 'tumour volume' (23.8% relative increase in hazard of death per doubling of 'tumour volume', 95% confidence interval 13.2–35.2%, $P<0.001$ stage I; $P<0.006$ stage II). In cases with stage IIIa tumours this factor alone was the significant prognostic variable. In conclusion, an estimate of 'tumour volume' significantly improves prediction of prognosis for individual NSCLC patients with UICC stage I or II tumours.

**Keywords:** lung cancer; 'tumour volume'; prognosis; survival

Non-small-cell lung carcinoma (NSCLC) accounts for about three-quarters of all lung cancer histologies. Surgical resection is the preferred treatment and approximately 50 000 operations are performed in the United States alone each year (Lederle and Neiwoehner, 1995). The overall prognosis for resected NSCLC is poor with less than a third of patients who undergo resection alive 5 years later (Humphrey *et al.*, 1990). There have been reports of many clinicopathological variables affecting prognosis of NSCLC. These have largely been examined in a univariate manner. They have included age, gender, TNM and other staging classifications, histopathological cell-types, oncogene expression and tumour biomarkers (Carney, 1992; Szabo and Mulshine, 1993).

In considering a large number of factors together multivariate analyses are valuable for determining which factors are independently influencing outcome and for categorising their relative importance. In patients with inoperable lung carcinoma multivariate anaalysis has shown the most important prognostic factors to be performance score, stage and weight loss in the previous 6 months (Stanley, 1980). In small-cell lung carcinoma proportional hazard models have shown performance status, age, gender and number of metastases to be the most significant predictors (Albain *et al.*, 1990; Gronowitz *et al.*, 1990).

We have previously shown that an estimate of NSCLC 'tumour volume' has a complex relationship with age which is dependent on patient gender and histological cell type (Pendleton *et al.*, 1996). In this study we examine the importance of 'tumour volume' as a predictor of survival after resection of NSCLC in a multivariate model including stage, patient age, gender, histopathological tumour cell type and nodal score.

Correspondence: N Pendleton
Received 9 November 1995; revised 12 February 1996; accepted 1 March 1996

### Materials and methods

The patient group comprised 669 cases with NSCLC treated by surgical resection between 1987 and 1992 at the Regional Thoracic Surgical Unit for Mersey region, UK. Patients were accepted solely on the basis of operability; age did not contribute to the assessment or selection process. All resections underwent thoracic nodal sampling at surgery.

Resected specimens were received in the department of histopathology at Broadgreen Hospital, Liverpool, UK, inflation-fixed in buffered formalin. The specimens were then examined macroscopically by a histopathologist whose examination included measurement of the maximum tumour diameter in three dimensions using a Vernier calliper. 'Tumour volume' was estimated by multiplying the three maximum dimensional measurements. Material from the specimens was taken from representative areas for subsequent light microscopic examination by two histopathologists within the department acting independently.

Each report generated from the department of histopathology included the following data: identification number, date of operation, date of birth, sex, specimens received, description of the macroscopic and microscopic findings including tumour site, dimensions, tumour cell type and nodal metastasis. Cell type was reported following the World Health Organization criteria (WHO, 1982) and nodal metastasis by the nodal score according to TNM classification criteria (UICC, 1987). Staging was performed according to the criteria proposed by Mountain *et al.* (1993). Cases of carcinoid, large-cell and mixed-cell-type tumours were excluded where analysis included cell type as a variable. An independent histopathological review of cell-type diagnosis was performed in a random group of 90 cases (13%).

For survival analysis, zero time for each subject was designated as the date of resection. The end point of the follow-up period was June 1994. Certified dates and causes of death were obtained from the National and Mersey and Cheshire Regional Cancer Registry.

Kaplan–Meier (product-limit) estimates were computed for the overall survival function unadjusted for any covariate factors. A more detailed analysis to investigate which clinical measures were related to (predictive of) survival time was then performed using Cox proportional hazards models (Cox, 1972). The Cox model is a semi-parametric regression model which can be stated in the form:

$$h_i(t) = h_0(t)\exp(\beta z)$$

Where $h_i(t)$ is the (proportional) hazard for the *i*th of *n* individuals at some time t, $\beta$ represents the regression coefficients for the matrix of independent predictor variables z, and $h_0$ is the hazard for an individual for whom all predictor variables have the value zero. Initially univariate analyses were done using a single clinical measure as the only predictor variable. Finally, a multivariate analysis was carried out in which survival predictor variables were allowed to enter the model in a forward stepwise manner; at each step an additional predictor variable being added if it significantly improved the prediction of survival. The level of significance for entry into the model was $P \leqslant 0.05$, and for removal was $P \geqslant 0.10$.

### Results

The distribution of the number of cases and 'tumour volume' by clinicopathological variable groups is summarised in Table I. The review of 90 randomly selected cases by an independent histopathologist blind to the original histopathological subtyping revealed disagreement in only one case.

Of the 669 subjects 12 were lost to follow-up. The length of follow-up ranged from 1 month to 80 months. In all 280 patients had died when the study was terminated. Cancer was certified as the cause of death in 246 of these. Overall the median survival was 66.0 months (95% confidence limits 50.6–81.4), the probability of survival at 2 years was 62.6% and at 5 years was 52.0%. The distribution of 'tumour volume' and probabilities of survival by stage, gender, cell-type and nodal score groups is shown in Table I (columns 2 and 3).

Univariate analysis of relationships between the variables described earlier by Cox proportional hazard is shown in Table II. Increasing risk of death was found to be significantly related to increased patient age at resection, male gender and increasing TNM nodal score. Tumour cell-type (adenocarcinoma compared with squamous cell carcinoma) was not significantly related to survival ($P = 0.33$).

Tumour stage and 'tumour volume' were both very significantly related to survival, there being an increased risk of death with increasing 'tumour volume' and stage group ($P < 0.001$).

Multivariate proportional hazard analysis involving the six variables found to be individually related to survival time

**Table I** Distribution of cases, 'tumour volume' and probabilities of survival at 2 and 5 years, by age, gender, TNM nodal score, tumour cell-type and UICC tumour stage after resection of NSCLC

| *Variable group* | *Number of cases* | *Mean 'tumour volume' ($cm^3$) with s.e.m.* | *Probability of survival (%) at 2 and 5 years* | |
|---|---|---|---|---|
| Gender | | | | |
| Male | 445 | 131.4±11.4 | 60.0 | 50.8 |
| Female | 224 | 78.4±11.5 | 68.0 | 54.9 |
| TNM nodal score | | | | |
| N0 | 434 | 108.0±10.9 | 71.5 | 60.0 |
| N1 | 115 | 115.2±15.5 | 50.6 | 44.7 |
| N2 | 120 | 123.0±16.1 | 41.8 | 29.7 |
| Tumour cell type | | | | |
| Adenocarcinoma | 230 | 114.6±16.5 | 59.4 | 47.7 |
| Squamous cell | 314 | 98.8±7.8 | 64.6 | 55.6 |
| Large cell | 44 | – | – | |
| Carcinoid | 22 | – | – | |
| Mixed cell types | 59 | – | – | |
| Stage | | | | |
| I | 403 | 91.6±8.6 | 73.2 | 60.8 |
| II | 106 | 92.4±13.0 | 53.4 | 45.0 |
| IIIa | 160 | 178.8±24.2 | 41.8 | 34.0 |

–, Calculations that were not made (see Materials and methods).

**Table II** Univariate Cox's proportional hazard analysis of relationship between age at resection, patient gender, TNM nodal score, UICC stage and 'tumour volume' after resection of NSCLC

| *Variables* | *Significance of relationship to survival time* | *Relative increase in hazard (95% CI)* | |
|---|---|---|---|
| Age at resection | $P = 0.027$ | 1.34% per year of increasing age | (0.1–2.5%) |
| Gender | $P = 0.024$ | 28.5% males *vs* females | (1.6–62.5%) |
| TNM nodal score | $P < 0.001$ | 85% N0 *vs* N1 | (35–152%) |
| | $P < 0.001$ | 151% N0 *vs* N2 | (89–233%) |
| Tumour cell type | $P = 0.330$ | – | |
| Tumour stage | $P < 0.001$ | 76.1% stage I *vs* II | (31.0–137%) |
| | $P < 0.001$ | 121% stage II *vs* III | (74.9–179%) |
| 'Tumour volume' | $P < 0.001$ | 19.2% per doubling of 'tumour volume' | (12.6–26%) |

indicated that, after taking account of the information provided by 'tumour volume' and stage in combination, age, gender, nodal score and tumour cell-type ceased to be predictive of survival. 'Tumour volume' and stage interacted significantly ($P<0.001$), indicating that the information provided by 'tumour volume' differed according to stage.

For stage I and II tumours, risk of death rose significantly as 'tumour volume' increased ($P<0.001$), the rate of increase being indistinguishable for the two stages ($P=0.54$); the risk of dying during a given period of time was increased by 23.8% (95% confidence limits 13.2–35.2%) with a doubling of tumour volume.

For stage IIIa tumours, 'tumour volume' was unrelated to survival ($P=0.44$), the fact that the tumour had reached this advanced stage being predictive of outcome alone. Relative to stage I and II tumour types, the risk of death associated with a stage III tumour was estimated as being increased by 485% (98% confidence limits 254–1250%).

Kaplan–Meier survival plots for stage I/II and IIIa tumour stages are shown in Figure 1.

**Figure 1** Survival functions of resected NSCLC patients grouped by tumour stage presented as Kaplan–Meier plots. Staging was performed using the criteria set out by Mountain (1993). □, stage IIIa; ○, stage II; ■, stage I.

### Discussion

Surgery is the treatment of choice for NSCLC although patients presenting with localised disease suitable for curative resection account for only 15% of all cases. For those treated with surgery, two-thirds will suffer recurrent disease within 5 years (Splinter, 1992; Martini, 1990; Shields, 1993). This has led to the examination of many clinicopathological variables that predict prognosis.

In clinical practice UICC stage (Mountain *et al.*, 1991) has emerged as the single most important criterion for management (Greenberg *et al.*, 1987). It is those cases with stage I, II or IIIa tumours that are generally considered suitable for surgery (Lederle and Neiwoehner, 1995). In common with other studies (Mountain, 1993) we have found stage to be an extremely significant independent predictor of survival, and to supplant other factors we examined in a multivariate model.

Within the TNM scale, the development of mediastinal metastasis has been shown to be a critical transition affecting survival (Wantanabe, 1991), and resectability (Mountain, 1993). This corresponds to the development of UICC stage IIIa from stage II. The results of this study support this finding, with no factor other than being in the stage IIIa group having prognostic significance by multivariate analysis in these cases.

Contrary to some reports (Rosenthal and Curran, 1990) we did not find tumour cell-type to be of prognostic significance. There may be three reasons for this difference. Firstly, while tumour cell-type and tumour stage relationships are reported (Teeter *et al.*, 1987; O'Rourke *et al.*, 1987) this does not imply an effect on survival.

Secondly, in this study comparison was only made between adenocarcinoma and squamous cell carcinoma, and unlike some previous studies, infrequent cell-type groups known to follow different courses such as large-cell and carcinoid tumours were excluded. Thirdly, as with all studies of measures generated from post-operative samples a selection bias may have occurred. The cases in this study may not necessarily be representative of the behaviour of the population of NSCLC tumours as a whole, and the results should rather be seen as representative only of those cases that have presented for and been selected for thoracic surgery.

We found age, male gender and nodal score to be univariately related to increased risk of death. However, these factors were supplanted by stage and 'tumour volume', and ceased to add further predictive information in the multivariate model. This would suggest that these factors are important in a population sense, but are not significant when considering an individual patient with a certain stage of tumour. The finding that transition from stage I or II to IIIa is of great prognostic significance, while TNM nodal score is not, indicates that this prognostic step involves more than mediastinal metastasis, and is related to the TNM tumour score–a function of which is tumour size. Lack of significance of 'tumour volume' with stage IIIa cases may, however, be caused by bias against larger tumours owing to incomplete resection.

A novel finding of this study is that 'tumour volume' is an extremely significant independent predictor of prognosis, and until the tumours have reached conditions for a classification of stage IIIa, appears to be the key factor, superseding the transition from stage I to II, age, gender, tumour cell-type and nodal metastasis.

We have previously shown that 'tumour volume' is related in a complex manner to age, gender and tumour cell-type (Pendleton *et al.*, 1996). The omission of these factors from the multivariate model with the inclusion of 'tumour volume' indicates that they may together act to influence prognosis by affecting the 'tumour volume'.

There has been much investigation of the prognostic significance of molecular factors (Carney, 1992; Szabo and Mulshine, 1993). While the inclusion of biomarker levels in a multivariate model has been shown to increase prognostic efficiency (Walop *et al.*, 1990), these techniques have not gained widespread clinical acceptance, are expensive and only available at a limited number of sites.

Thus, we conclude that while UICC staging remains the gold standard in prognosis and management of NSCLC, this study shows that an estimate of 'tumour volume', an easy-to-calculate measure that requires no specialist equipment, gives significant prognostic information over and above that of the UICC stage. We therefore suggest that estimation of 'tumour volume' should be considered as part of the routine post-operative prognostic work-up of NSCLC. Multivariate analyses including other factors such as oncogene expression and histopathological measures together with 'tumour volume' in prognosis of NSCLC merit further investigation.

**Acknowledgement**
We would like to acknowledge the help of the Mersey and Cheshire Regional Cancer Registry.

### References

ALBAIN KS, CROWLEY JJ, LEBLANC M AND LIVINGSTONE RB. (1980). Determinants of improved outcome in small-cell lung cancer: an analysis of the 2580-patient Southwest Oncology Group Data Base. *J. Clin. Oncol.*, **8,** 1563-1574.

CARNEY DN. (1992). The biology of lung cancer. *Curr. Opin. Oncol.*, **4,** 292-298.

COX DR. (1972). Regression models and life-tables, and discussion. *J. R. Stat. Soc. B.*, **34,** 187-202.

GREENBERG SD, FRAIRE AE, KINNER DM AND JOHNSON EH. (1987). Tumour cell type versus staging in the prognosis of carcinoma of the lung. *Pathol. Ann.*, **22,** 387-405.

GRONOWITZ JS, BERGSTROM R, NOU E, PAHLMAN S, BRODIN O, NILSSON S AND KALLANDER GFR. (1990). Clinical and serologic marker of stage and prognosis in small cell lung cancer. *Cancer*, **66,** 722-732.

HUMPHREY EW, SMART CR, WINCHESTER DP, STEELE GD JR, YARBRO JW, CHU KC AND TRIOZO MM. (1990). National survey of the pattern of care for carcinoma of the lung. *J. Thorac. Cardiovasc. Surg.*, **100,** 837-843.

LEDERLE FA AND NEIWOEHNER, DE. (1995). Lung cancer surgery, a critical review of the evidence. *Arch. Intern. Med.*, **154,** 2397-2400.

MARTINI N. (1990). Surgical treatment of lung cancer. *Semin. Oncol.*, **17,** 9-10.

MOUNTAIN CF. (1993). Lung cancer stage in classification. *Clin. Chest. Med.*, **14,** 43-53.

MOUNTAIN CF, GREENBERG SD AND FRAIRE AE. (1991). Tumour stage in non-small cell carcinoma of the lung. *Chest*, **99,** 1258-1260.

O'ROURKE MA, FEUSSNER JR, FEIGL P, LASZIO J. (1987). Age trends of lung cancer stage at diagnosis. Implications for lung cancer screening in the elderly. *JAMA*, **258,** 921-926.

PENDLETON N, JEFFERSON MF, DIXON GR, MYSKOW MW AND HORAN MA. (1996). Correlates of tumour size and metastasis of resected non-small cell lung cancer (NSCLC) with age. *J. Gerontol.*, **51A,** B50-B53.

ROSENTHAL SA AND CURRAN WJ JR. (1990). The significance of histology in non-small cell lung cancer. *Cancer Treat. Rev.*, **17,** 409-425.

SHEILDS TW. (1993). Surgical therapy for carcinoma of the lung. *Clin. Chest. Med.*, **14,** 121-147.

SPLINTER TAW. (1992). Therapy for small cell and non-small cell lung cancer. *Curr. Opin. Oncol.*, **4,** 315-322.

STANLEY KE. (1980). Prognostic factors for survival in patients with inoperable lung cancer. *J. Natl Cancer Inst.*, **65,** 25-32.

SZABO E AND MULSHINE J. (1993). Epidemiology, prognostic factors, and prevention of lung cancer. *Curr. Opin. Oncol.*, **5,** 302-309.

TEETER SM, HOLMES FF AND MCFARLANE MJ. (1987). Lung carcinoma in the elderly population. Influence of histology on the inverse relationship of stage to age. *Cancer*, **60,** 1331-1336.

UICC (INTERNATIONAL UNION AGAINST CANCER). (1987). *TNM Classification of Malignant Tumours.* 4th ed. Hermanek P, Sobin LH (eds). Springer-Verlag: Berlin.

WALOP W, CHRETIEN, M, COLMAN NC, FRASER RS, GILBERT F, HIDVEGI RS, HUTCHINSON T, KELLY B, LIS M, SPITZER WO AND SUISSA S. (1990). The use of biomarkers in the prediction of survival in patients with pulmonarycarcinoma. *Cancer*, **65,** 2033-2046.

WANTANABE Y, HAYASHI Y, SHIMIZU J, ODA M AND IWA T. (1991). Mediastinal nodal involvement and the prognosis of non-small cell lung cancer. *Chest*, **100,** 422-428.

WORLD HEALTH ORGANIZATION. (1982). The World Health Organization histological typing of lung tumours. *Am. J. Clin. Pathol.*, **77,** 123-136.